# ChatGPT-5 versus other mainstream large language models in core diabetic retinopathy patient queries

**DOI:** 10.3389/fcell.2026.1754221

**Published:** 2026-03-25

**Authors:** Xiaomin Cang, Mengxia Ni, Chunyan Song, Jialuo Zhao, Yingxin Guo, Yunyun Zou, Zhe Zhang, Ligang Jiang

**Affiliations:** 1 Department of Endocrinology, Nantong First People’s Hospital, Southeast University, Nantong, China; 2 Hai’an High-tech District Central Health Center, Nantong, Jiangsu, China; 3 Shenzhen Eye Hospital, Shenzhen Eye Medical Center, Southern Medical University, Shenzhen, China; 4 Department of Ophthalmology, Quzhou People’s Hospital, Quzhou Affiliated Hospital of Wenzhou Medical University, Quzhou, Zhejiang, China

**Keywords:** accuracy and safety, artificial intelligence, diabetic retinopathy, large language models, ophthalmic digital health, patient-initiated consultation

## Abstract

**Background:**

Diabetic retinopathy is a leading cause of preventable vision loss, and patients increasingly seek disease related information through online consultations. Large language models may support patient education, but their reliability and usability vary across systems, particularly in disease specific settings.

**Methods:**

Thirty common patient questions about diabetic retinopathy were developed from guidelines and organized into five domains: disease overview, screening and diagnosis, treatment and follow up, lifestyle and prevention, and prognosis and complication management. From November 10 to 15, 2025, two researchers independently submitted all questions to five models (ChatGPT-5, DeepSeek-V3.1, Doubao, Wenxinyiyan 4.5 Turbo, and Kimi) on public platforms under identical conditions without system prompts. Chat histories were reset before each question. Response time, response length, structural metrics, and table outputs were extracted. Two retinal specialists rated each answer on a 1 to 5 Likert scale across accuracy, logical consistency, coherence, safety, and content accessibility. Inter rater agreement was assessed with the intraclass correlation coefficient. Group differences were analyzed using analysis of variance or the Kruskal–Wallis H test with Bonferroni corrected pairwise comparisons.

**Results:**

Significant between model differences were observed in output efficiency and textual characteristics (all P < 0.001). ChatGPT-5 responded fastest (15.92 ± 4.48 s), whereas Wenxinyiyan 4.5 Turbo and DeepSeek-V3.1 were slowest (41.89 ± 5.09 s and 38.20 ± 2.96 s). DeepSeek-V3.1 generated the longest answers (1396.37 ± 189.23 words), while Kimi produced the shortest (579.40 ± 182.96 words). Only ChatGPT-5 consistently generated structured tables (median 2.00, IQR 1.00-2.00). Content quality differed significantly across all five dimensions (H = 15.34-37.19, all P ≤ 0.004). ChatGPT-5 achieved the highest median scores for accuracy (5.00, IQR 4.00-5.00) and logical consistency (4.50, IQR 4.00-5.00), whereas Kimi showed the lowest accuracy (3.50, IQR 3.00-4.00). The intraclass correlation coefficient indicated good inter rater reliability (0.87).

**Conclusion:**

Performance of large language models in diabetic retinopathy patient consultations is model dependent. ChatGPT-5 demonstrated the best overall usability, combining faster responses, clearer structure, and higher factual accuracy. Other Chinese optimized models provided comparable professional information coverage but require improved accessibility and stability for safe patient facing use.

## 1 Introduction

Diabetic retinopathy (DR) is one of the most common microvascular complications of diabetes and a leading cause of preventable vision impairment among working-age adults worldwide, with a particularly high risk of blindness (Modjtahedi et al., 2020; Simó and Hernández, 2023). A recent international meta-analysis estimated that approximately 22.3% of people with diabetes have DR, including about 6.2% with vision-threatening DR. In 2020, the global number of individuals with DR was about 103 million and is projected to rise to about 160 million by 2045 (Teo et al., 2021). With the continuing increase in diabetes prevalence and the accelerating trend of population aging, the burden of DR is expected to grow further (Hou et al., 2023). Although early screening, standardized treatment, and patient education have been proven to substantially reduce the risk of vision loss (Waheed et al., 2023), DR screening coverage and the level of patient health education remain inadequate globally (Lundeen et al., 2023; Yu et al., 2025).

In recent years, rapid advances in artificial intelligence (AI) (Chen et al., 2020; Jiang et al., 2025; Ting et al., 2018), particularly the emergence of large language models (LLMs), have created new opportunities for medical information dissemination, health education, and digital healthcare services (Wei et al., 2025; Li et al., 2025). Trained on massive corpora, LLMs can understand and generate natural language and are capable of simulating expert-level responses to health consultations (Wei et al., 2024; Heinke et al., 2024; Huang et al., 2025). Prior studies have shown that LLMs hold promise in clinical decision support, patient education, medical literature summarization, and teleconsultation; however, concerns persist regarding imperfect medical factuality, hallucinated outputs, potential biases, and limited interpretability (Wu et al., 2024; Zhang Q. et al., 2025).

Within the specific context of DR, patients frequently seek online or intelligent question and answer systems for guidance on screening intervals, treatment options, lifestyle interventions, and prognosis management. Addressing this clinical need, the present study focuses on DR, the most common blinding complication of diabetes, and evaluates model performance in disease education and patient consultation. By comparing multiple LLMs using a multidimensional, quantitative expert rating framework, this work aims to provide evidence for future AI-assisted patient education and public health communication, and to explore the feasibility and safety of LLMs in retinal disease management.

## 2 Materials and methods

### 2.1 Study design

**FIGURE 1:**
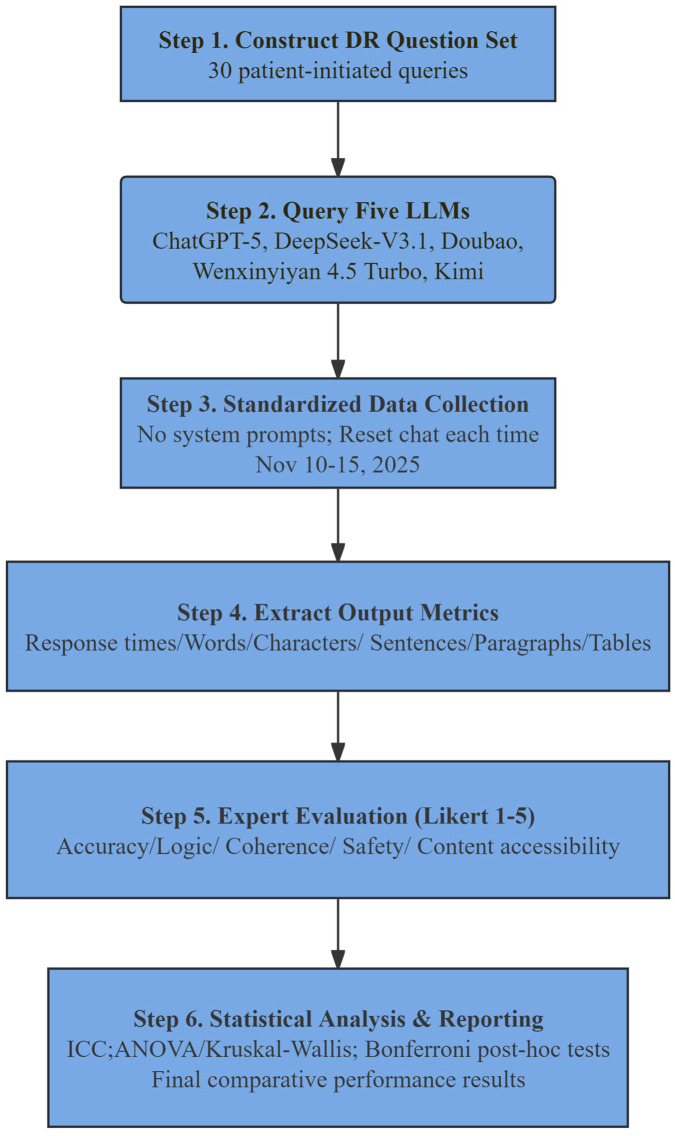
Study workflow for benchmarking five LLMs on DR-related patient queries. Two researchers queried five LLMs using a standardized 30-question set (Chinese, single-turn, single-shot) and extracted response efficiency/structure metrics (response times, length and structural indicators). Two retinal specialists independently rated each response on a 1–5 Likert scale across five dimensions (accuracy, logic, coherence, safety, and content accessibility). Each model generated 30 responses (n = 30 per model; total responses = 150), and each response received two expert ratings (total ratings = 300). Statistical comparisons followed the prespecified parametric/non-parametric pipeline with multiplicity-adjusted *post hoc* tests.

A cross-sectional comparative design was used to systematically evaluate performance differences among five mainstream LLMs in answering patient-initiated DR-related questions. The study workflow comprised six steps: construction of a question set, model response generation, standardized data collection, extraction of output indicators, expert scoring, and statistical analysis (Figure 1). All models generated answers independently under identical conditions to ensure comparability and reproducibility.

### 2.2 LLMs selection

#### 2.2.1 GPT-5 (OpenAI)

OpenAI’s flagship next-generation model released in August 2025. It employs a unified-routing dual-mode system (“fast response” plus “deep reasoning [thinking]”), automatically adjusting reasoning depth to task complexity, thereby reducing hallucinations and improving reliability in medical and coding scenarios. GPT-5 achieved 74.9% on SWE-bench Verified and supports up to about 400K-token long-context input, enabling stronger context retention for complex, multi-turn medical consultations (Handler et al., 2025). For this study, the model was accessed via its official web interface at https://chatgpt.com.

#### 2.2.2 DeepSeek-V3.1

An open-weight hybrid-reasoning Mixture-of-Experts (MoE) model released by DeepSeek in August 2025 (671B total parameters, 37B activated per token). It supports Think and Non-Think mode switching, 128K long-context extension training, and dedicated optimization for Chinese tasks, offering both performance and efficiency in complex reasoning and long-form question answering (Sandmann et al., 2025). The model was queried via the public web platform at https://chat.deepseek.com.

#### 2.2.3 Doubao

A Chinese general-purpose LLM system continuously upgraded by ByteDance throughout mid-2025, with large-scale deployment across multiple industries. Public reports indicate daily token usage exceeding 16.4 trillion, reflecting strong Chinese dialogue and multi-turn interaction capabilities. It was included as a representative widely used Chinese application-oriented model (Liu, 2025). We utilized the web version available at https://www.doubao.com.

#### 2.2.4 Wenxinyiyan 4.5 turbo

An efficient version of Baidu’s ERNIE 4.5 series released at Create 2025. While retaining ERNIE 4.5’s multimodal understanding, coding, and logical reasoning strengths, it further optimizes inference latency and cost, and reduces hallucination risk via knowledge and retrieval augmentation strategies, making it highly practical for Chinese-language applications (Kang et al., 2025). We utilized the web version available at https://yiyan.baidu.com.

#### 2.2.5 Kimi (moonshot AI, kimi K2)

An open-weight MoE model released by Moonshot AI in July 2025 (about 1T total parameters, 32B activated). It provides long-context capability and strong code generation and agentic skills, performing well on SWE-bench Verified (about 65.8%) and optimized for Chinese scenarios. It was included as a representative domestic long-context and coding-capable model (Gibney, 2025). Access was obtained through the web interface at https://kimi.moonshot.cn.

### 2.3 Construction of the question set

Thirty patient-initiated DR questions were developed through a two-stage, guideline-anchored expert consensus process to support content coverage and face validity for patient counseling. First, two ophthalmologists independently extracted candidate questions from authoritative DR guidelines and high-impact reviews (including Standards of Care in Diabetes-2025 and the American Academy of Ophthalmology Preferred Practice Pattern for DR) (Diabetes Care in the Hospital, 2025; Lim et al., 2025), targeting core patient-education themes and key management decision points commonly encountered in routine practice (e.g., definition/staging, screening intervals, treatment options, self-management, prognosis, and warning signs of complications). Second, the clinician panel met to merge duplicates, refine wording, and consolidate items through iterative review informed by recurring topics observed in routine DR outpatient consultations and patient education, while ensuring balanced coverage across five predefined domains (disease overview; screening and diagnosis; treatment and follow-up; lifestyle and prevention; prognosis/complication management). Discrepancies were resolved by discussion with a senior retinal specialist, and the final set was checked for domain coverage, clarity, and patient-facing readability. To approximate real-world online consultations, all items were rewritten in a patient-voice style with minimal medical jargon while preserving the original clinical intent.

### 2.4 Data collection procedure

Between November 10 and 15, 2025 (time zone: China Standard Time, UTC+8), two researchers independently queried each LLM using the standardized 30-question set via the models’ publicly accessible web interfaces. All queries were performed within a consistent daily time window (09:00–18:00) to reduce variability due to diurnal fluctuations in server load. For each question, a new chat session was initiated and the history was manually cleared to avoid carryover effects. All models were queried in a single-turn, single-shot setting without additional system prompts to capture default patient-facing behavior under minimal user intervention. Each question was submitted once as a standalone query, without follow-up turns, additional patient-history supplementation, emotional cues, or conversational steering; therefore, this protocol does not aim to reproduce the full complexity of real-world patient consultations. For each response, we extracted response time, word/character counts, sentence and paragraph numbers, and the presence of tabular outputs. All models responded in Chinese for all queries. Because length metrics are language-dependent and word count is not directly comparable across languages, we treated character count as the primary length indicator for Chinese responses. Textual metrics (including word/character counts, sentence and paragraph numbers) were computed using TextLen with Chinese segmentation enabled, and tabular outputs were manually counted by two researchers.Response time was recorded as end-to-end user-perceived latency on the web interface (from question submission to completion of the initial full response). Because such measurements are influenced by network latency, geographic routing, server load, and platform infrastructure, response time should be interpreted as environment-specific, user-perceived latency rather than intrinsic model inference speed. To reduce variability, all measurements were performed using the same device, browser, and network connection within the defined collection window, following an identical timing procedure. All raw responses and extracted metrics were double-checked and stored in a shared Microsoft Excel file for subsequent scoring and statistical analysis.

### 2.5 Performance evaluation

Two attending vitreoretinal ophthalmologists (each with 5 years of clinical experience) independently rated all responses. Prior to scoring, all answers were de-identified and randomly coded so that raters were blinded to model identity. Each response was evaluated on a 5-point Likert scale across five dimensions. To minimize subjectivity and ensure verifiable metrics, the Accuracy dimension was strictly anchored to the specific clinical guidelines used for question generation (e.g., Standards of Care in Diabetes-2025 and the American Academy of Ophthalmology Preferred Practice Pattern for DR) (Diabetes Care in the Hospital, 2025; Lim et al., 2025). A score of 5 (Excellent) required the model’s response to be fully concordant with the guideline recommendation without errors; a score of 3 (Acceptable) indicated general alignment but with minor omissions; and scores ≤2 indicated contradiction with established medical consensus. Other dimensions included logical consistency (internal reasoning and conclusion coherence), coherence (clarity and organization of narrative), safety (objectively assessed based on the presence of necessary referral advice), and content accessibility (patient-friendly language and actionability). Any disagreements ≥2 points were resolved through discussion to reach a consensus score for analysis. Inter-rater reliability was quantified using intraclass correlation coefficients (ICCs).

### 2.6 Statistical analysis

All analyses were performed in SPSS (version 27.0). Inter-rater reliability for expert scoring was assessed using ICCs Continuous output metrics were tested for normality using the Shapiro–Wilk test and for homogeneity of variance using Levene’s test; normally distributed variables are presented as mean ± standard deviation(SD), whereas non-normal variables are reported as median (IQR). Likert-scale content quality ratings were treated as ordinal data and are presented as median (IQR). Comparisons among the five models were conducted using one-way ANOVA for normally distributed continuous variables and the Kruskal–Wallis H test for ordinal or non-normal variables. When the overall test was significant, Bonferroni-adjusted *post hoc* pairwise comparisons were performed (*post hoc* tests after ANOVA; pairwise Mann–Whitney U tests following Kruskal–Wallis). All tests were two-sided, and p < 0.05 was considered statistically significant.

## 3 Results

### 3.1 Comparison of output efficiency and textual characteristics among five mainstream LLMs

As shown in Table 1, the five LLMs differed significantly in response times, answer length, and structural richness (all P < 0.001). Regarding output efficiency, ChatGPT-5 had the shortest response time (15.92 ± 4.48 s), significantly faster than DeepSeek-V3.1 (38.20 ± 2.96 s), Doubao (27.07 ± 2.91 s), Wenxinyiyan 4.5 Turbo (41.89 ± 5.09 s), and Kimi (34.41 ± 2.37 s). For text length, DeepSeek-V3.1 generated the longest responses (1396.37 ± 189.23 words; 1612.37 ± 241.06 characters), whereas Kimi produced the shortest (579.40 ± 182.96 words; 729.37 ± 251.06 characters). ChatGPT-5 yielded intermediate lengths (833.53 ± 168.01 words; 994.10 ± 203.30 characters). Similar trends were observed for sentences and paragraphs: DeepSeek-V3.1 had the highest sentence count (71.17 ± 11.05), ChatGPT-5 had the highest paragraph count (46.27 ± 6.76), and Kimi had the lowest counts for both sentences (27.17 ± 5.94) and paragraphs (16.40 ± 5.83). Notably, only ChatGPT-5 consistently produced tabular content (2.00, IQR 1.00-2.00), while tabular output from the other models was near zero.

**TABLE 1:** Comparison of output efficiency and textual characteristics across five LLMs.

Metric	ChatGPT-5	DeepSeek-V3.1	Doubao	Wenxinyiyan 4.5 Turbo	Kimi	P-value
Response times	15.92 ± 4.48	38.20 ± 2.96	27.07 ± 2.91	41.89 ± 5.09	34.41 ± 2.37	**<0.001**
Words	833.53 ± 168.01	1396.37 ± 189.23	785.50 ± 189.58	1006.97 ± 209.84	579.40 ± 182.96	**<0.001**
Characters	994.10 ± 203.30	1612.37 ± 241.06	829.13 ± 205.54	1062.30 ± 230.18	729.37 ± 251.06	**<0.001**
Sentences	57.30 ± 8.02	71.17 ± 11.05	36.87 ± 4.93	44.33 ± 11.51	27.17 ± 5.94	**<0.001**
Paragraphs	46.27 ± 6.76	36.97 ± 6.91	29.50 ± 2.54	32.57 ± 9.29	16.40 ± 5.83	**<0.001**
Tables	2.00(1.00,2.00)	0.00(0.00,0.00)	0.00(0.00,1.00)	0.00(0.00,0.00)	0.00(0.00,1.00)	**<0.001**

Data for response times, words, characters, sentences, and paragraphs followed a normal distribution and are presented as Mean ± SD., Data for tabular outputs followed a non-normal distribution and are presented as Median (Q1, Q3). Overall group differences were tested using one-way ANOVA, or the Kruskal–Wallis H test as appropriate. Bold values indicate statistical significance after adjustment (*P* < 0.05).

**FIGURE 2:**
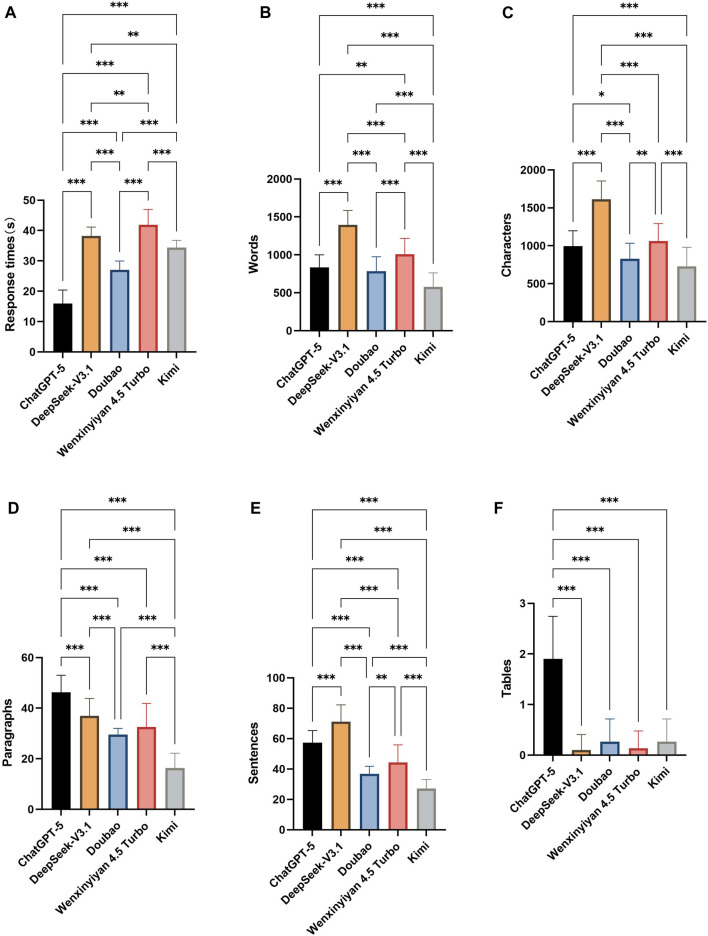
Response efficiency and structural characteristics across the five LLMs. Pairwise comparisons for: **(A)** Response times; **(B) **Words; **(C)** Characters; **(D)** Sentences; **(E)** Paragraphs; **(F)** Tables. Overall group differences were assessed using one-way ANOVA or Kruskal–Wallis H test based on normality; pairwise comparisons were Bonferroni-adjusted. Significance notation: *P < 0.05, **P < 0.01, ***P < 0.001, Absence of asterisks indicates no statistical significance. Sample size n = 30 queries per model (Total N = 150).

Pairwise comparisons are detailed in Table 2 and Figure 2. ChatGPT-5 was significantly faster than all other models in response time (all P < 0.001). For textual features, ChatGPT-5 generated significantly fewer words than DeepSeek-V3.1 and Wenxinyiyan 4.5 Turbo (P < 0.001 and P = 0.004, respectively), but did not differ from Doubao (P = 0.860). ChatGPT-5 produced fewer characters than DeepSeek-V3.1 (P < 0.001), with no significant difference versus Wenxinyiyan 4.5 Turbo (P = 0.770). Structurally, ChatGPT-5 generated significantly more paragraphs, sentences, and tables than the other models (all P < 0.001). DeepSeek-V3.1 produced significantly more words and characters than Doubao, Wenxinyiyan 4.5 Turbo, and Kimi (all P < 0.001). Except for ChatGPT-5, differences in table output among the remaining models were not significant (P = 1.000).

**TABLE 2:** Pairwise comparisons of output efficiency and textual structure metrics among five LLMs.

Comparison	Response times	Words	Characters	Paragraphs	Sentences	Tables
ChatGPT-5 vs. DeepSeek-V3.1	**<0.001**	**<0.001**	**<0.001**	**<0.001**	**<0.001**	**<0.001**
ChatGPT-5 vs. Doubao	**<0.001**	0.860	**0.040**	**<0.001**	**<0.001**	**<0.001**
ChatGPT-5 vs. Wenxinyiyan 4.5 Turbo	**<0.001**	**0.004**	0.770	**<0.001**	**<0.001**	**<0.001**
ChatGPT-5 vs. Kimi	**<0.001**	**<0.001**	**<0.001**	**<0.001**	**<0.001**	**<0.001**
DeepSeek-V3.1 vs. doubao	**<0.001**	**<0.001**	**<0.001**	**<0.001**	**<0.001**	1.000
DeepSeek-V3.1 vs. Wenxinyiyan 4.5 Turbo	**0.002**	**<0.001**	**<0.001**	0.080	**<0.001**	1.000
DeepSeek-V3.1 vs. Kimi	**0.001**	**<0.001**	**<0.001**	**<0.001**	**<0.001**	1.000
Doubao vs. Wenxinyiyan 4.5 Turbo	**<0.001**	**<0.001**	**<0.001**	0.380	**0.010**	1.000
Doubao vs. Kimi	**<0.001**	**<0.001**	0.440	**<0.001**	**<0.001**	1.000
Wenxinyiyan 4.5 Turbo vs. Kimi	**<0.001**	**<0.001**	**<0.001**	**<0.001**	**<0.001**	1.000

Overall group differences were tested using one-way ANOVA, or the Kruskal–Wallis H test as appropriate. All *post hoc* pairwise *P*-values are Bonferroni-adjusted for multiple comparisons. Bold values indicate statistical significance after adjustment (*P* < 0.05).

### 3.2 Comparison of five-dimension content-quality scores among five mainstream LLMs

**FIGURE 3:**
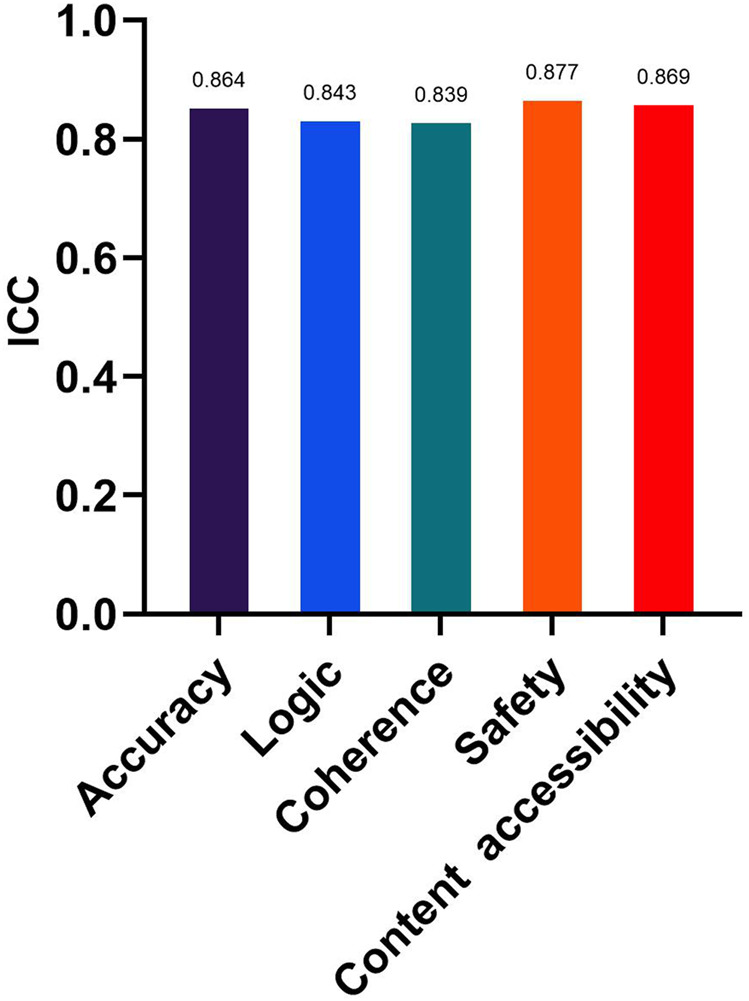
Inter-rater reliability of expert Likert ratings. ICCs for the five evaluation dimensions (accuracy, logic, coherence, safety, content accessibility). ICCs are reported with 95% confidence intervals. Two retinal specialists rated all responses independently (two-way random-effects model, absolute agreement).

Figure 3 presents the ICCs between the two retinal specialists for response scoring on accuracy, logic, coherence, safety, and content accessibility. All ICCs indicated good to excellent agreement (range 0.839–0.877), supporting the reliability of the evaluation process. As shown in Table 3, significant between-model differences were observed across all five dimensions (H = 15.34-37.19, all P ≤ 0.004). ChatGPT-5 achieved the highest median scores for accuracy (5.00, IQR 4.00-5.00) and logic (4.50, IQR 4.00-5.00). Kimi had the lowest overall ratings, particularly for accuracy (3.50, IQR 3.00-4.00). Coherence and safety remained generally high across models (median mostly 4.00), though ChatGPT-5 showed a more favorable distribution, yielding significant overall differences. Content accessibility was also highest for ChatGPT-5 (4.00, IQR 4.00-5.00).

**TABLE 3:** Comparison of five LLMs across five dimensions of content quality.

Metric	ChatGPT-5	DeepSeek-V3.1	Doubao	Wenxinyiyan 4.5 Turbo	Kimi	H-value	ε^2^	P-value
Accuracy	5.00(4.00,5.00)	4.00(3.75,4.00)	4.00(4.00,4.00)	4.00(3.00,4.00)	3.50(3.00,4.00)	37.19	0.229	**<0.001**
Logic	4.50(4.00,5.00)	4.00(3.00,4.00)	4.00(3.00,4.00)	4.00(3.00,4.00)	4.00(2.75,4.00)	29.62	0.177	**<0.001**
Coherence	4.00(4.00,5.00)	4.00(3.00,4.00)	4.00(4.00,4.25)	4.00(3.00,4.25)	4.00(2.75,4.00)	17.37	0.092	**0.002**
Safety	4.00(4.00,4.25)	4.00(3.00,4.00)	4.00(4.00,4.00)	4.00(3.00,4.00)	4.00(3.00,4.00)	15.34	0.078	**0.004**
Content accessibility	4.00(4.00,5.00)	4.00(3.75,4.00)	4.00(3.00,4.00)	4.00(3.00,4.00)	4.00(3.00,4.00)	26.44	0.155	**<0.001**

Overall group differences were tested using the Kruskal–Wallis H test k=5,n=150 . Effect size was reported as epsilon-squared (ε^2^), calculated as ε^2^ = (H − k + 1)/(n − k). Bold values indicate statistical significance (*P* < 0.05).

**FIGURE 4:**
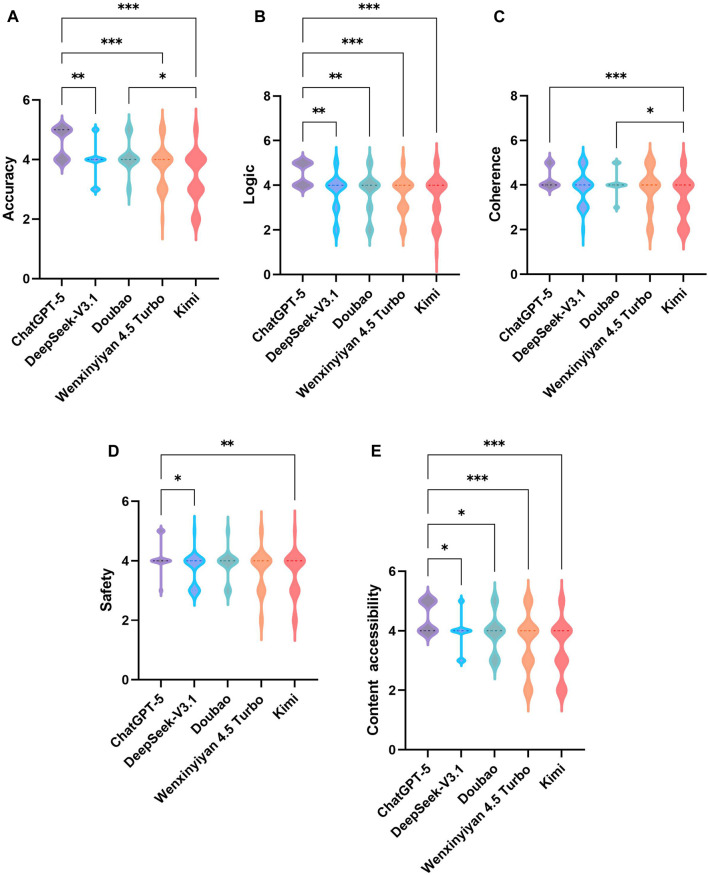
Content-quality Likert ratings across the five LLMs. Pairwise comparisons for: **(A)** accuracy, **(B)** logic, **(C)** coherence, **(D)** safety, **(E)** content accessibility on a one to five Likert scale. Each model contributed 30 responses, rated by two specialists (n = 60 ratings per model per dimension). Plots display distribution/variance (e.g., median/IQR). Group differences were tested using Kruskal–Wallis H tests, followed by Bonferroni-adjusted pairwise comparisons. Significance notation: *P < 0.05, **P < 0.01, ***P < 0.001, Absence of asterisks indicates no statistical significance.

Post hoc pairwise comparisons are shown in Table 4 and Figure 4. ChatGPT-5 scored significantly higher than DeepSeek-V3.1 on accuracy, logic, coherence, safety, and content accessibility (P = 0.004- 0.028), although the difference in coherence was not significant (P = 0.283). Compared with Doubao, ChatGPT-5 performed better in logic and content accessibility (P = 0.002 and 0.028), with no significant differences in other dimensions (P ≥ 0.084). ChatGPT-5 also outperformed Wenxinyiyan 4.5 Turbo in accuracy, logic, and content accessibility (all P ≤ 0.001). Versus Kimi, ChatGPT-5 was significantly superior in all five dimensions (all P ≤ 0.005). Differences among DeepSeek-V3.1, Doubao, and Wenxinyiyan 4.5 Turbo were not significant across dimensions (all P = 1.000). Doubao scored higher than Kimi in accuracy and coherence (P = 0.013 and 0.037).

**TABLE 4:** Pairwise comparisons of content quality dimensions among the five LLMs.

Comparison	Accuracy	Logic	Coherence	Safety	Content accessibility
ChatGPT-5 vs. DeepSeek-V3.1	**0.004**	**0.001**	0.283	**0.022**	**0.028**
ChatGPT-5 vs. Doubao	0.084	**0.002**	1.000	0.536	**0.028**
ChatGPT-5 vs. Wenxinyiyan 4.5 Turbo	**<0.001**	**<0.001**	0.452	0.056	**0.001**
ChatGPT-5 vs. Kimi	**<0.001**	**<0.001**	**0.001**	**0.005**	**<0.001**
DeepSeek-V3.1 vs. doubao	1.000	1.000	1.000	1.000	1.000
DeepSeek-V3.1 vs. Wenxinyiyan 4.5 Turbo	1.000	1.000	1.000	1.000	1.000
DeepSeek-V3.1 vs. Kimi	0.217	1.000	0.745	1.000	0.721
Doubao vs. Wenxinyiyan 4.5 Turbo	1.000	1.000	1.000	1.000	1.000
Doubao vs. Kimi	**0.013**	1.000	**0.037**	1.000	0.718
Wenxinyiyan 4.5 Turbo vs. Kimi	0.914	1.000	0.483	1.000	1.000

Overall group differences were tested using the Kruskal–Wallis H test. All *post hoc* pairwise *P*-values are Bonferroni-adjusted for multiple comparisons. Bold values indicate statistical significance after adjustment (*P* < 0.05).

## 4 Discussion

Using 30 core DR-related consultation questions, this study compared five mainstream LLMs across multiple dimensions in a patient health-consultation setting. Overall, ChatGPT-5 achieved the highest median scores in accuracy, logic, and content accessibility. DeepSeek-V3.1, Doubao, and Wenxinyiyan 4.5 Turbo formed a second-tier group with broadly comparable performance, whereas Kimi ranked lowest, particularly in accuracy. Significant between-model differences were observed across all five dimensions, indicating that the clinical usability of LLMs for patient-facing DR consultations remains heterogeneous (Antaki et al., 2024; Wang et al., 2025; Srinivasan et al., 2025).A clear advantage of ChatGPT-5 in accuracy and logic was observed, aligning with prior findings that stronger general-purpose models outperform smaller or local models in ophthalmic clinical scenarios. Huang et al. (Huang et al., 2024) reported that GPT-4 class models generally surpassed other LLMs in diagnostic reasoning and treatment recommendations for ophthalmologic scenarios, though instability and hallucinations persisted for certain sub-tasks. Mechanistically, the superior performance of ChatGPT-5 in Accuracy and Logic (Median 5.0 and 4.5) likely stems from its underlying unified-routing dual-mode system (Handler et al., 2025). Unlike standard dense models, this architecture allows for dynamic allocation of inference-time compute, effectively engaging “System 2” thinking (deep reasoning) for complex medical causality queries while using fast paths for simple definitions. This architectural advantage minimizes the hallucination bottleneck often seen in purely autoregressive generation, thereby ensuring higher logical consistency in explaining DR complications. In contrast, while DeepSeek-V3.1 and Kimi utilize MoE architectures to maximize parameter efficiency and context length (Sandmann et al., 2025; Gibney, 2025), their lower accuracy scores (3.50–4.00) suggest that without specific system prompts, their routing mechanisms may prioritize fluency over strict medical factuality in zero-shot settings.The present data further suggest that the upgraded ChatGPT-5 integrates high factual accuracy, transparent reasoning, and patient-friendly wording more effectively for common DR questions, implying generational improvements in training scale, RLHF, safety alignment, and medical instruction tuning. Notably, David et al. (Mikhail et al., 2025) found that DeepSeek-R1 outperformed OpenAI o1 in diagnostic accuracy and management appropriateness for ophthalmic case analyses. Although based on earlier model versions, this evidence underscores the possibility of task- and scenario-dependent advantages among models, highlighting the need for multi-model, dynamic, real-world validation within specific diseases and use cases. Furthermore, our findings cross-validate and extend recent benchmarks in the field. Wu et al. (2025) reported that ChatGPT-4.0 achieved about 97.6% favorable accuracy ratings in DR patient Q&A and held a slight edge in accuracy and safety. Our study confirms that this high-performance trajectory is maintained and potentially refined in GPT-5, particularly in its ability to spontaneously generate structured tables—a capability less prominent in earlier iterations. However, a divergence is noted when comparing Chinese models: while Wu et al. (2025) found significant safety gaps in earlier models, our results show that current Chinese LLMs (Doubao, Wenxinyiyan) have achieved parity with ChatGPT-5 in Safety scores (Median 4.0). This indicates that while the reasoning gap (Accuracy/Logic) persists due to differences in pre-training corpora and reinforcement learning from human feedback (RLHF) strategies, the safety gap is rapidly closing due to rigorous alignment efforts in the Chinese distinct research ecosystem. Here, DeepSeek-V3.1, Doubao, and Wenxinyiyan 4.5 Turbo demonstrated comparable coverage and professionalism, yet this did not translate into higher accuracy or content accessibility scores. These findings support the notion that longer, more professional-looking responses are not necessarily more reliable or easier to understand, potentially due to redundant information, high terminology density, and insufficient contextual explanation (Gotlieb et al., 2022; Lang et al., 2025). DeepSeek-V3.1 generated longer responses and required more time, but its quality did not significantly exceed that of Doubao or Wenxinyiyan 4.5 Turbo. In contrast, ChatGPT-5, while more concise, received the highest overall evaluation. This aligns with Jung et al. (Jung et al., 2024), who noted that optimized prompting or alignment strategies can enhance key-point coverage and readability in retinal disease Q and A, whereas unconstrained verbosity may reduce patient comprehension efficiency (Zhang Z. et al., 2025). Thus, the current results support a clinically grounded perspective: the value of patient-facing LLMs lies not in producing more text, but in correctly covering essential information, presenting it in an understandable manner, and clearly delineating risk boundaries (Huo et al., 2025; Sharma et al., 2025).

The observed performance advantage of ChatGPT-5 may be attributable to differences in medical instruction-tuning, safety alignment, and robustness of reasoning under uncertainty, although these explanations remain hypothesis-generating rather than definitive (Singhal et al., 2025; Farquhar et al., 2024). Betzler et al. (2023) emphasized that hallucinations, guideline lag, and insufficient risk messaging are key bottlenecks for ophthalmic LLM applications, and that real-world deployment must be grounded in structured evaluation and safety alignment. Although median safety scores were high across models, ChatGPT-5 was more consistent in advising medical follow-up, communicating risk boundaries, and avoiding over-commitment, consistent with stronger safety training and refusal policies. Meanwhile, DeepSeek-V3.1, Wenxinyiyan 4.5 Turbo, and Doubao offered competitive professional coverage, indicating promising potential for Chinese LLMs in medical corpora and localized knowledge integration, but with room for improvement in patient-oriented accessibility and stability control (Griot et al., 2025). Crucially, a qualitative analysis of our safety data reveals a divergence between procedural safety and content safety. While all models achieved high Safety scores by consistently advising medical consultation, this metric masks underlying risks. Models with lower Accuracy or Logic scores (e.g., Kimi, DeepSeek-V3.1) exhibited a latent safety hazard: they provided appropriate warnings yet simultaneously generated factually incorrect medical explanations or logical contradictions. For example, a response might correctly advise seeing a doctor but incorrectly explain the mechanism of vision loss, potentially causing patient confusion or anxiety. Therefore, true patient safety in LLMs requires not just the presence of risk flags, but the strict elimination of plausible-sounding hallucinations. However, a critical linguistic asymmetry must be addressed. This study utilized Chinese-only queries, theoretically favoring domestic models, which possess architectures and training corpora explicitly optimized for Chinese linguistic nuances and cultural contexts. In contrast, ChatGPT-5, while multilingual, is primarily trained on English-centric data. Surprisingly, the expected “home-field advantage” of domestic models in language fluency did not translate into superior medical accuracy or logical reasoning. This discrepancy suggests that the reasoning gap in core medical knowledge between ChatGPT-5 and domestic models currently outweighs the linguistic gap. ChatGPT-5’s superior performance in a non-primary language underscores its robust cross-lingual generalization capabilities, whereas Chinese-native models may require further alignment in medical reasoning to match their linguistic proficiency.Overall, this study provides quantitative evidence for Chinese-language DR LLM interactions: at present, LLMs may serve as auxiliary tools for health education and preliminary triage, but their eligible populations and application boundaries require careful differentiation (Robinson et al., 2024).

An additional observation was that ChatGPT-5 more frequently used tables or highly structured bullet-point formats when responding to patient questions. This style likely reflects enhanced instruction-following and structured-expression capability in newer models. Recent medical-domain LLM studies suggest that instruction tuning and RLHF encourage models to organize complex information into scannable, verifiable structured units, thereby improving usability and safety (Xie et al., 2025; Yan et al., 2025). Consistently, Van Veen et al. (2024) found that structured, itemized, or quasi-tabular answers facilitate rapid verification of key information and reduce omissions or misunderstanding in clinical text summarization tasks. Therefore, ChatGPT-5’s structure-first, explanation-second pattern may contribute to its superior readability and information coverage.

However, tabular output can be a double-edged sword. On one hand, tables compress and highlight key comparative dimensions, helping patients grasp core frameworks, an effect also noted by Freyer et al. (2024), who advocated for more actionable structured interfaces in future LLM-based health applications. On the other hand, overly regularized tables may conceal inherent medical uncertainties or prerequisites, visually amplifying perceived authority and fostering over-trust. Harrer (2023) cautioned that highly polished formats such as clear tables and bullet lists may enhance readability while also magnifying the persuasiveness of incorrect information. Taken together, these findings suggest that ChatGPT-5’s tabular strength is well suited for public education and pre-decision information organization, yet caution is warranted for complex queries requiring individualized, context-rich explanations where oversimplification may occur.

Several limitations should be acknowledged. First, responses were collected within a single, time-stamped window with one query per question; given stochastic generation and rapid model iteration, model stability and temporal drift cannot be fully characterized, and repeated sampling across releases is warranted, ideally using multi-center and multi-time-point verification. Second, the standardized Chinese-only, single-turn protocol improves comparability but does not reflect real-world consultations involving multi-turn follow-up, emotional reassurance, iterative history supplementation, or heterogeneous questioning styles across health-literacy levels; thus, generalizability to broader populations and multilingual settings is limited. Third, although the 30-item question bank was guideline-anchored and refined through clinician consensus informed by routine outpatient counseling, it was not frequency-validated via patient interviews/surveys or mining real-world consultation data, leaving potential “doctor-perspective vs. patient-needs” bias. Fourth, content quality relied on expert Likert ratings by two specialists; despite acceptable agreement, residual rater bias and partial unblinding from model-specific formatting cannot be excluded, and subjective ratings may not translate to real patient comprehension or behavioral change. Fifth, only text-based Q&A was assessed; multimodal scenarios (e.g., fundus image interpretation) and a systematic taxonomy of harmful outputs (hallucinations, contradictions, or hazardous recommendations) were not fully evaluated. Accordingly, conclusions mainly apply to the tested web-interface versions for Chinese-language basic DR counseling and chronic-management triage; LLMs should not be used to counsel potential acute complications (e.g., sudden vision loss, eye pain, or new flashes/floaters), which require urgent in-person evaluation, and broader real-world deployments require prospective validation.

Because LLMs are iteratively updated, our findings should be interpreted as a time-stamped benchmark (Nov 10–15, 2025) rather than a permanent ranking. Nevertheless, the durable contribution of this work is the clinician-grounded evaluation blueprint: a domain-structured DR question taxonomy, a multi-dimensional scoring rubric (accuracy, logic, safety boundaries, and accessibility), and a standardized public web-interface testing workflow in Chinese. The safety-relevant failure modes highlighted here (e.g., inconsistent red-flag escalation and internally contradictory advice) are model-agnostic targets that remain informative across model versions. Future studies should treat this benchmark as updateable via repeated sampling across releases and extensions to multi-turn and multilingual settings.

## 5 Conclusion

Substantial differences were identified among five mainstream LLMs in response efficiency and content quality for DR-related patient queries. ChatGPT-5 demonstrated the best overall performance in response speed, structured expression, and the dimensions of accuracy, logic, and content accessibility. DeepSeek-V3.1, Doubao, and Wenxinyiyan 4.5 Turbo showed broadly similar but slightly inferior performance, whereas Kimi was relatively weaker, particularly in accuracy. These findings support a clinically grounded view that the value of patient-facing LLMs depends less on producing longer responses and more on correctly and clearly covering key points, using accessible language, and explicitly defining risk boundaries. At present, LLMs demonstrate the foundational content quality to serve as auxiliary tools for DR health education and preliminary triage, rather than stand-alone clinical decision-makers. Specifically, effective preliminary triage should be strictly limited to chronic management inquiries; questions involving acute red flag symptoms must trigger immediate referral protocols rather than LLMs counseling. Regarding model optimization, Chinese-developed systems should prioritize logic-first alignment and structured brevity over textual length, bridging the gap between their strong professional coverage and clinical usability.

## Data Availability

The original contributions presented in the study are included in the article/supplementary material, further inquiries can be directed to the corresponding authors.
